# PReS-FINAL-2216: Biomarkers MRP8/14 and S100A12 correspond with flare and remission clinical status in Sojia patients in the AID-NET register

**DOI:** 10.1186/1546-0096-11-S2-P206

**Published:** 2013-12-05

**Authors:** F Gohar, E Husmann, PJ Haas, G Horneff, H Wittkowski, D Holzinger, T Kallinich, U Neudorf, T Niehues, E Lainka, D Foell

**Affiliations:** 1 Department of Paediatric Rheumatology and Immunology, University of Muenster, Muenster, Germany; 2 University Children's Hospital, Children's Unit, Paediatric Rheumatology, Essen, Germany; 3 German Center for Child and Adolescent Rheumatology, Garmisch-Partenkirchen, Garmisch-Partenkirchen, Germany; 4 Center of General Paediatrics and Neonatology, Asklepios Clinic Sankt Augustin GmbH, Sankt Augustin, Germany; 5 Department of Pediatric Pneumology and Immunology, Department of Rheumatology, Charité - Universitätsmedizin Berlin, Berlin, Germany; 6 Paediatric Rheumatology, University of Duisburg-Essen, Essen, Germany; 7 Center for Child and Adolescent Medicine, HELIOS Hospital Krefeld, Krefeld, Germany; 8 Paediatric Rheumatology, University Hospital Essen, Children's Hospital, Essen, Germany

## Introduction

Systemic onset juvenile idiopathic arthritis (SoJIA) shows properties of autoinflammatory disease, and requires the presence of arthritis and fever for diagnosis. The pro-inflammatory proteins MRP8/14 (S100A8/9) and S100A12 are biomarkers which have been shown to detect ongoing subclinical disease activity in patients with clinical remission. We compared the MRP8/14 (S100A8/9) and S100A12 biomarker profile of patients in the AID-NET (German Auto-inflammatory Disease Network) register to further provide evidence for the usefulness of measurement of these biomarkers.

## Objectives

To characterize levels of pro-inflammatory markers of innate immune activation, S100A12 and MRP8/14, during active and inactive disease status of patients with SoJIA.

## Methods

The AID-Net register, which includes patients with SoJIA diagnosed according to ILAR criteria, was searched for patients with clinically defined remission and flare episodes. Patients in remission were those documented as being either: 'non-acute', in remission, or with a clinician score of <1 (range 0-10, where 0 represents inactivity and 10 represent highly active disease). Flares were any cases scored as 'flare' or 'acute'. Statistical significance was measured using the Mann-Whitney-U test, using SPSS.

## Results

55 patients with a median age of 13 years (range 5-20 years) at time of blood sampling were included. A total of 158 episodes occurred where disease activity status and MRP8/14 and S100A12 results were available. This included 38 episodes of flares (19 patients) and 120 episodes of remission (44 patients). Patients presenting with episodes of flare had significantly higher mean S100A12 values compared with patients in remission (mean 2,895 (range 15-19,410) ng/ml vs 575 (0-6,220) ng/ml, respectively, p- < 0.01). MRP 8/14 values were also higher in patients who were clinically flaring than in those in remission (9,600 (100-48,610) ng/ml vs 2,965 (0-45,390) ng/ml, p < 0.01).

## Conclusion

The measurement of MRP8/14 and S100A12 biomarkers in patients with SoJIA in the AID-net register corresponded well with the recorded clinical disease activity in these patients. This provides further evidence for the measurement of these biomarkers.

References: 1) Foell D et al. Methotrexate withdrawal at 6 vs 12 months in juvenile idiopathic arthritis in remission: a randomized clinical trial. JAMA 2010;303:1266-73. 2) Gerss J et al. Phagocyte-specific S100 proteins and high-sensitivity C reactive protein as biomarkers for a risk-adapted treatment to maintain remission in juvenile idiopathic arthritis: a comparative study. Ann Rheum Dis 2012;71:1991-7.

## Disclosure of interest

None declared.

